# Venetoclax with low-dos**e** cytarabine, a forgotten combination in patients with acute myeloid leukemia ineligible for intensive chemotherapy: a systematic review

**DOI:** 10.1016/j.htct.2024.07.006

**Published:** 2024-09-23

**Authors:** Lauro Fabián Amador-Medina, Erick Crespo-Solís, Francisco Javier Turrubiates-Hernández, Karla Edith Santibañez-Bedolla

**Affiliations:** a Clinical Epidemiology Research Unit, OOAD Guanajuato, Mexican Social Security Institute, Leon, Guanajuato, Mexico; b Department of Medicine and Nutrition, University of Guanajuato, Leon, Guanajuato, Mexico; c Christus Muguerza Hospital Faro del Mayab, Merida, Yucatan, Mexico; d Institute of Research in Biomedical Sciences, University Center of Health Sciences, University of Guadalajara, Guadalajara, Jalisco, Mexico

**Keywords:** Acute myeloid leukemia, Venetoclax, Azacitidine, Low-dose cytarabine, Ineligible for intensive chemotherapy

## Abstract

**Background:**

Based on the VIALE-A and VIALE-C studies, the Food and Drug Administration approved venetoclax in 2020 in combination with azacitidine or low-dose cytarabine for the treatment of patients with acute myeloid leukemia ineligible for intensive chemotherapy. After the publication of these studies, venetoclax/azacitidine was assumed to be superior to venetoclax/low-dose cytarabine; however, these studies were not designed to demonstrate superiority between these combinations. Therefore, we conducted a systematic review to describe overall survival, complete remission rate, and composite complete remission rate to assess response of these two regimens in patients with newly diagnosed acute myeloid leukemia who are ineligible for intensive chemotherapy.

**Materials and methods:**

The PubMed and Web of Science databases were searched for retrospective studies and complete remission, composite complete remission, and overall survival rates were recorded.

**Results:**

Only 11 of the 815 publications identified were eligible to be included n this review, ten studies evaluated the venetoclax/azacitidine combination and one study evaluated the venetoclax/low-dose cytarabine combination. The median overall survival for venetoclax/azacitidine was 10.75 months, whereas for venetoclax/low-dose cytarabine the median overall survival had not been reached at the time of publication. Composite complete remission was 63.3 % for venetoclax/azacitidine and 90 % for venetoclax/low-dose cytarabine. Adverse events were similar for both combinations.

**Conclusions:**

A limited number of studies investigating the venetoclax/low-dose cytarabine combination exist. Based on the available data, the superiority of venetoclax/azacitidine over venetoclax/low-dose cytarabine cannot be assumed for all acute myeloid leukemia patients who are ineligible for intensive chemotherapy. Venetoclax/low-dose cytarabine can still be considered as an option for the drug combinations currently under investigation.

## Introduction

Intensive chemotherapy is considered the treatment of choice for newly diagnosed patients with acute myeloid leukemia (AML). However, not all patients are eligible for this treatment because of physical limitations, comorbidities, or advanced age.^1^ Based on the results of two international, multicenter clinical trials (VIALE-A and VIALE-C),^2^^,^^3^ in 2020 the U.S. Food and Drug Administration (FDA)^4^ approved treatment with venetoclax (VEN) in combination with azacitidine (VEN/AZA) or low-dose cytarabine (VEN/LDAC) in over 75-year-old patients with newly diagnosed AML or in patients ineligible for intensive chemotherapy as induction therapy. In these trials, median overall survival (OS) was 14.7 months for the VEN/AZA combination versus 8.4 months for the VEN/LDAC combination.^2^^,^^3^

In the daily practice, hematologists often must choose between VEN/AZA and VEN/LDAC when treating AML patients who cannot receive intensive chemotherapy.

Based on the results of the VIALE-A^2^ and VIALE-C^3^ trials, the medical community has generally assumed that the VEN/AZA combination is superior to the VEN/LDAC combination in the treatment of these patients. However, assuming the superiority of one regimen over the other based solely on the results of these two clinical trials has several major limitations. The first and perhaps the most important limitation is that these trials aimed not to determine whether VEN/AZA is superior to VEN/LDAC. The second most important limitation is that the VIALE-C trial included a higher proportion of patients with secondary AML (41 %)^3^ compared to the VIALE-A trial (25 %);^2^ a factor often associated with worse OS curves.

Recent research has focused on evaluating the effect of VEN in various combinations (fludarabine, cladribine, and others) and with other indications such as myelodysplastic syndrome, relapsed or refractory AML or even first-line intensive chemotherapy,^5^, ^6^, ^7^, ^8^, ^9^, ^10^ while the option of LDAC has lagged behind as a therapeutic option.

To our knowledge, there has been no formal comparison to categorically demonstrate the superiority of VEN/AZA, especially in patients with intermediate and favorable cytogenetic prognosis. This is particularly important in Latin American countries with low-income populations. It has already been pointed out that VEN/AZA is unlikely to be accessible to the entire population because of its current cost.^11^

For these reasons, we conducted a systematic review of real-world studies to describe OS, complete remission rate (CR), and composite complete remission rate (CCR) to assess response to the VEN/AZA or VEN/LDAC combinations in patients with newly diagnosed AML ineligible for intensive chemotherapy.

## Materials and methods

The search strategy, study selection, and data synthesis were based on the 2020 version of the Preferred Reporting Items for Systematic Reviews and Meta-Analyses (PRISMA) guidelines.^12^ The protocol was submitted to PROSPERO (https://www.crd.york.ac.uk/prospero/) and was assigned the registration number CRD4202022367290. With the original search criteria, we did not find enough clinical trials with the VEN/LDAC combination to perform a meta-analysis. Therefore, we amended the protocol, updating it with the same registration number (CRD4202022367290). Therefore, the present study corresponds to a systematic review of the current literature, including only the analysis of retrospective studies with real-world experience to describe the main outcomes (OS, CR, CCR) associated with VEN/AZA and VEN/LDAC regimens.

### Eligibility criteria and data sources

To determine the effect of the VEN/AZA and VEN/LDAC combinations, we considered retrospective studies with real-world experience conducted in populations with characteristics similar to those of the VIALE-A^2^ and VIALE-C^3^ studies (e.g. newly diagnosed, previously untreated, and ineligible for intensive chemotherapy). This review included studies that specifically allowed extraction of data for the VEN/AZA and VEN/LDAC combinations in the non-pretreated population, regardless of whether the studies had a comparison group, as the results of the comparison group were not considered for this review.

A more detailed description of the inclusion and exclusion criteria established for screening articles is provided in Table S1 (Supplementary Material).

In October and November 2022, we performed a comprehensive search of the PubMed and Web of Science electronic databases. We reviewed studies in Spanish and English available from January 1, 2015 to September 30, 2022.

### Search strategy and selection process

The search strategy was performed using the combination of the following keywords in the title and abstract: ‘retrospective study’, ‘retrospectively’, ‘cohort’, ‘observational’, ‘acute myeloid leukemia’, ‘AML’, ‘azacitidine’, ‘azacytidine’, ‘vidaza’, ‘cytarabine’, ‘cytosar’, ‘aracytine’, ‘venetoclax’, and ‘venclexta’. The search string is presented in more detail in Table S2 of the supplementary material.

The results of the search in each database were loaded into the Rayyan software.^13^ Two reviewers independently reviewed the titles and abstracts and selected the studies that met the eligibility criteria for full-text review. The two reviewers discussed disagreements and if a consensus could not be reached, a third reviewer was consulted.

### Data collection process

Two reviewers independently extracted information from the included studies and collated the data, which was entered into a spreadsheet containing the variables of interest. If discrepancies were found, they were resolved in collaboration with a third reviewer. Finally, all authors participated simultaneously in the revision of the database.

### Data items

The review focused only on outcomes of interest from patients treated with VEN/AZA or VEN/LDAC who had not received prior treatment. The outcomes of interest in each study were the OS, CR, and CCR rates according to internationally established definitions.^1^ Other data collected were the cytogenetic risk, somatic mutations, and Grade 3 or higher adverse events according to the Common Terminology Criteria for Adverse Events (CTCAE).

For studies that reported OS in days, this was converted to months (days of follow-up/30). Valid percentages were calculated for the variables of interest based on the information provided in the articles reviewed (text and tables). Similarly, medians were determined as summary measures, with their dispersion values (minimum and maximum) in parentheses. The Statistical Package for Social Sciences (SPSS) version 25.0 (IBM, Chicago, IL, USA) was used.

### Risk of bias assessment

The risk of bias was assessed using the Newcastle-Ottawa Scale (NOS) to assess the studies included in this systematic review.^14^ The NOS includes eight items to assess study quality derived from three domains (selection of study groups, comparability of groups, and determination of exposure or outcome of interest - Table S3). The assessment was conducted independently by two reviewers and in case of disagreement, a third reviewer was consulted.

## Results

### Selection of studies

**Figure 1:**
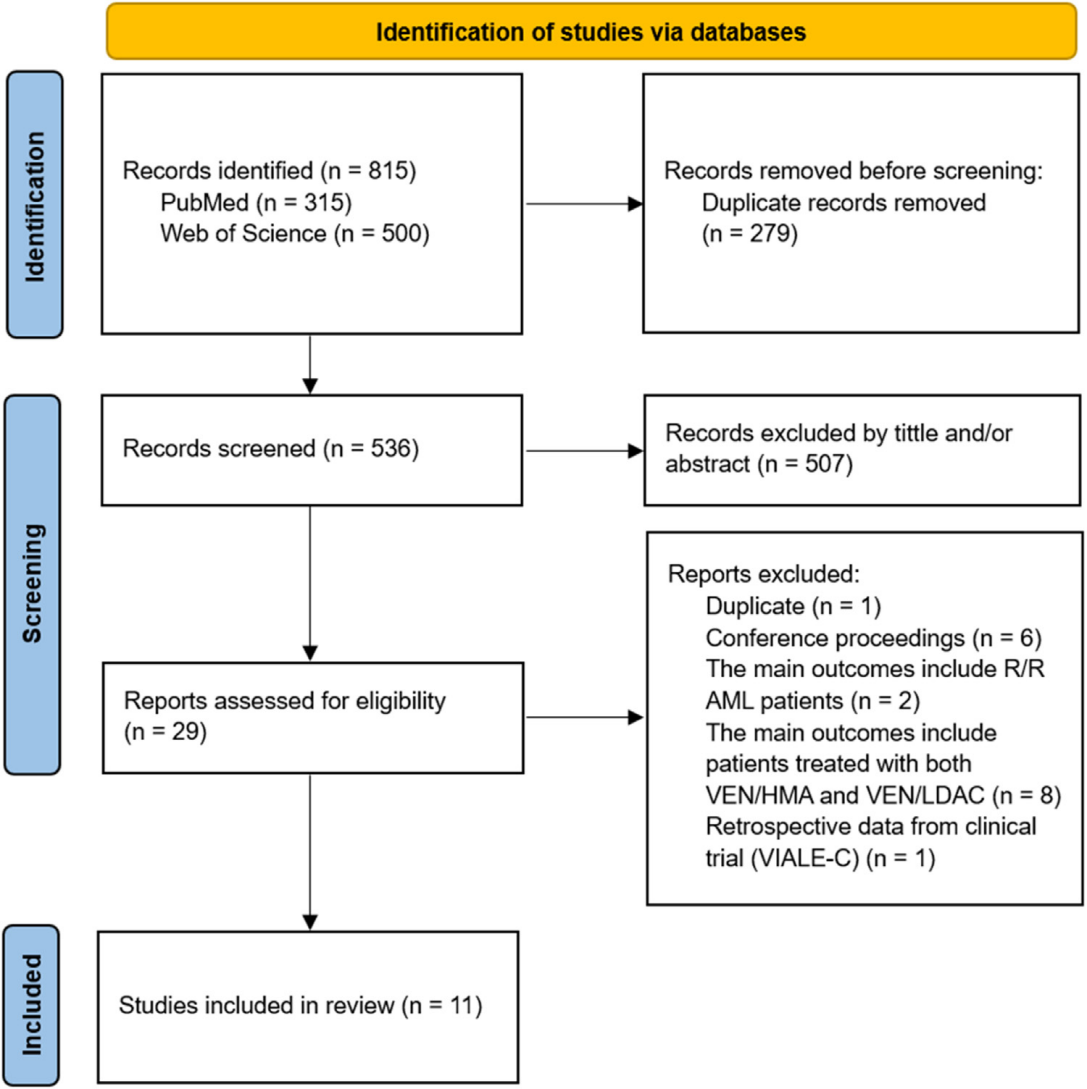
PRISMA 2020 flow diagram of the selection of studies included in the systematic review. Abbreviations: R/R AML, relapsed or refractory acute myeloid leukemia; VEN, venetoclax; HMA, hypomethylating agents (azacitidine or decitabine); LDAC, low-dose cytarabine.

Our search yielded a total of 815 abstracts. Of these, 315 (38.7 %) were found in PubMed and 500 (61.3 %) in the Web of Science. We excluded 279 duplicate abstracts before the screening phase. Subsequently, 507 articles (94.6 %) were excluded based on the title and/or abstract, as they were mainly conference proceedings, letters to the editor, preclinical reports, literature reviews, and clinical trials. The studies VIALE-A^2^ and VIALE-C^3^ were only included in the discussion section due to their prospective design. Therefore, 29 (5.6 %) articles were selected for full-text review. Based on the inclusion criteria, another 18 articles were excluded. Most of these exclusions were because they were reports of refractory or relapsed cases or because the results were mixed with other drug combinations. Finally, 11 studies of newly diagnosed patients were included: ten on the effects of VEN/AZA in patients with AML ineligible for chemotherapy,^15^, ^16^, ^17^, ^18^, ^19^, ^20^, ^21^, ^22^, ^23^, ^24^ and only one study that addressed both VEN/AZA and VEN/LDAC, with information collected separately.^25^ The flow diagram of this study identification is shown in Figure 1.

### Risk of bias assessment

According to the NOS, the median score of the studies was 5 stars (range: 4–9 stars). Four (36.4 %) of the studies were classified as good quality with a median of 8 stars (range: 8–9 stars).^17^^,^^20^^,^^21^^,^^23^ The remaining seven studies (63.6 %) were considered fair with a median of 5 stars (range: 4–6 stars).^15^, ^16^^,^^18^, ^19^^,^^22^^,^^25^ None of the studies were of poor quality. Table S3 provides details of the risk of bias assessment.

### Characteristics of included studies

The retrospective studies in this review were published between 2019 and 2022. Of the 11 included studies, seven (63.6 %) were conducted in the United States,^15^, ^16^, ^17^^,^^20^^,^^21^^,^^23^^,^^24^ one in India,^18^ one in Italy,^19^ one in France,^22^ and one in Mexico-Peru.^25^ Five (45.4 %) of the studies were multicenter^19^, ^20^, ^21^^,^^24^^,^^25^ and six (54.6 %) were conducted at a single center.^15^, ^16^, ^17^, ^18^^,^^22^^,^^23^ A sample of 1023 patients with newly diagnosed AML who were ineligible for intensive chemotherapy was obtained from all studies, of whom, 1013 (99 %) were treated with the VEN/AZA regimen, while only ten (1 %) received the VEN/LDAC combination.

Of the studies where the distribution by sex could be determined, 370 (46.1 %) of the patients were female and 433 (53.9 %) were male; however, they were all treated with the VEN/AZA regimen.^16^^,^^17^^,^^20^, ^21^, ^22^, ^23^ In the only study examining the VEN/LDAC combination, it was not possible to determine the distribution by sex as this variable was reported in association with patients receiving VEN/AZA.^25^ The median age of the patients was 72 years (range: 69.5–75 years), a datum that was only reported in cases that received the VEN/AZA regimen.^15^, ^16^, ^17^^,^^21^, ^22^, ^23^ Table 1 provides an overview of the characteristics of the 11 studies.

**Table 1:** Characteristics of the 11 retrospective studies included in the systematic review.

First Author, Year	Study Design, Population	VEN/AZA and/or VEN/LDAC	Number of Patients, Sex (*n*)	Median Age, Years (range)	Secondary AML (%) b	Median Follow-up, Months (95 % CI)	CR Rate (%)	CCR Rate (%)	OS Median, Months (95 % CI)
Winters et al., 2019	Single-center, USA	VEN/AZA	30 (F: ND/M: ND)	72 (ND)	36.7	8.5 (3.2–12.1)	43.3	63.3	12.7 (5.8-not reached)
Abbott et al., 2020	Single-center, USA	VEN/AZA	101 (F: 54/M: 47)	72 (22–89)	ND	24.9 (6.3–218.7)	13.8	65.3	ND
Cherry et al., 2021	Single-center, USA	VEN/AZA	143 (F: 71/M: 72)	69.5 (22–91)	41.3	26.9 (ND)	62.2	71.3	16.1 (ND)
Mirgh et al., 2021	Single-center, India	VEN/AZA	16 (F: ND/M: ND)	≥30 a	ND	8 (ND)	ND	75	ND
De Bellis et al., 2021	Multi-center, Italy	VEN/AZA	43 (F: ND/M: ND)	≥50 a	ND	9.9 (1.2–32.5)	48.8	67.4	10.5 (ND)
Jensen et al., 2022	Multi-center, USA	VEN/AZA	58 (F: 25/M: 33)	≥60 a	ND	ND	37.5	46.4	9.1 (3.4–11.9)
Matthews et al., 2022	Multi-center, USA	VEN/AZA	439 (F: 191/M: 248)	75 (36–88)	48	8.8 (ND) c	17	43	11 (ND)
Garciaz et al., 2022	Single-center, France	VEN/AZA	38 (F: 17/M: 21)	73 (61–81)	35	ND	32	45	9.4 (ND)
Mustafa Ali et al., 2022	Single-center, USA	VEN/AZA	24 (F: 12/M: 12)	70.5 (67.2–78.4)	50	20.53 (18.2–26.47)	ND	58.3	12.3 (9.2-not reached)
Vachhani et al., 2022	Multi-center, USA	VEN/AZA	103 (F: ND/M: ND)	≥18 a	ND	ND	ND	50	ND
Gómez-De León et al., 2022	Multi-center, Mexico and Peru	VEN/AZA	18 (F: ND/M: ND)	≥16 a	ND	ND	ND	72.2	9.6 (4.2–15)
Gómez-De León et al., 2022	Multi-center, Mexico and Peru	VEN/LDAC	10 (F: ND/M: ND)	≥16 a	ND	ND	ND	90	Not reached

VEN: venetoclax; AZA: azacitidine; LDAC: low-dose cytarabine; F: female; M: male; AML: acute myeloid leukemia; CR: complete remission; CCR: composite complete remission; OS: overall survival; ND: not determined.

a Age stated in the selection criteria since age is not specified for the group treated with the VEN/AZA or VEN/LDAC combination.

b Sum of the rates of treatment-related acute myeloid leukemia and antecedent hematologic disorder.

c Mean.

### Overall survival

Eight of the 11 studies that treated patients with the VEN/AZA combination reported OS. The median OS was 10.75 months (range: 9.1–16.10 months).^15^^,^^17^^,^^19^, ^20^, ^21^, ^22^, ^23^^,^^25^ In contrast, the median OS was not reached in the only study in which the VEN/LDAC combination was administered.^25^ The median follow-up time reported in seven of the 11 studies was 9.9 months (range: 8–26.9 months); however, this was exclusively for the VEN/AZA regimen (Table 1).^15^, ^16^, ^17^, ^18^, ^19^^,^^21^^,^^23^

### Complete remission and composite complete remission

The CR rate was reported in seven studies that used the VEN/AZA combination with a median CR of 37.5 % (range: 13.8–62.2 %).^15^, ^16^, ^17^^,^^19^, ^20^, ^21^, ^22^ CCR rates were reported in all 11 studies of the VEN/AZA combination, with a median of 63.3 % (range: 43–75 %).^15^, ^16^, ^17^, ^18^, ^19^, ^20^, ^21^, ^22^, ^23^, ^24^, ^25^ In contrast, the only study that used the VEN/LDAC combination did not report the CR rate, however the CCR rate was 90 %.^25^ Table 1 summarizes the CR and CCR rates for each study.

### Risk categories

All studies reported cytogenetic risk categories based on the 2017 European Leukemia Network (ELN) risk classification.^26^ A total of 94 patients (13.5 %) with favorable cytogenetic risk, 200 (28.8 %) with intermediate risk, and 401 (57.7 %) with adverse cytogenetic risk were reported.^15^, ^16^, ^17^^,^^20^, ^21^, ^22^, ^23^

Overall, favorable, intermediate, and adverse cytogenetic risk rates occurred in 12.5 % (range: 4.5–23.3 %), 27.3 % (range: 10–86.4 %), and 53.3 % (range: 9.1–66.7 %), respectively, of patients treated with VEN/AZA.^15^, ^16^, ^17^, ^18^, ^19^, ^20^, ^21^, ^22^, ^23^, ^24^ In seven of the ten studies, the most common risk category was adverse,^15^, ^16^, ^17^^,^^20^, ^21^, ^22^^,^^24^ whereas, in the remaining studies, the intermediate category predominated.^18^^,^^19^^,^^23^ The study by Gómez-De León et al., (combined VEN/AZA and VEN/LDAC) reported frequencies of 10.7 %, 21.4 %, and 25 % ^25^ for the favorable, intermediate, and adverse risk categories, respectively (Table 2).

**Table 2:** Frequencies of response risks and somatic mutations of the 11 retrospective studies included in the systematic review.

First Author, Year	VEN/AZA and/or VEN/LDAC	Risk Categories, *n* (%) a			Somatic Mutations, *n* (%)							
		Favorable	Intermediate	Adverse	*FLT3* ITD	*IDH1*	*IDH2*	*IDH 1–2*	*ASXL1*	*RUNX1*	*NPM1*	*TP53*
Winters et al., 2019	VEN/AZA	7/30 (23.3)	3/30 (10)	20/30 (66.7)	5/30 (16.7)	–	–	5/30 (16.7)	11/30 (36.7)	–	–	5/30 (16.7)
Abbott et al., 2020	VEN/AZA	22/99 (22.2)	18/99 (18.2)	59/99 (59.6)	–	–	–	–	–	–	–	–
Cherry et al., 2021	VEN/AZA	24/141 (17)	24/141 (17)	93/141 (66)	20/143 (14)	15/143 (10.5)	24/143 (16.8)	39/143 (27.3)	36/143 (25.3)	23/143 (16.2)	33/143 (23.1)	25/143 (17.4)
Mirgh et al., 2021 b	VEN/AZA	3/24 (12.5)	15/24 (62.5)	6/24 (25)	3/14 (21.4)	2/14 (14.2)	–	–	–	2/14 (14.2)	6/14 (42.8)	–
De Bellis et al., 2021 c	VEN/AZA	–	28/51 (54.9)	23/51 (45.1)	8/54 (14.8)	–	–	9/54 (16.6)	–	–	10/54 (18.5)	3/7 (42.8)
Jensen et al., 2022	VEN/AZA	6/58 (10.3)	19/58 (32.8)	33/58 (56.9)	–	–	–	–	–	–	–	–
Matthews et al., 2022	VEN/AZA	34/323 (10.5)	117/323 (36.2)	172/323 (53.3)	30/321 (9.3)	–	–	72/316 (22.8)	42/329 (12.8)	29/329 (8.8)	36/313 (11.5)	57/329 (17.3)
Garciaz et al., 2022	VEN/AZA	–	–	22/38 (57.9)	2/38 (5.3)	7/38 (18.4)	1/38 (2.6)	8/38 (21)	14/30 (46.6)	10/30 (33.3)	3/38 (7.9)	8/35 (22.9)
Mustafa Ali et al., 2022	VEN/AZA	1/22 (4.5)	19/22 (86.4)	2/22 (9.1)	2/23 (8.7)	–	–	–	4/17 (23.5)	5/17 (29.4)	2/17 (11.8)	3/17 (17.6)
Vachhani et al., 2022 d	VEN/AZA	22/169 (13)	37/169 (21.9)	65/169 (38.5)	–	–	–	–	–	–	–	–
Gómez-De León et al., 2022 e	VEN/AZA and VEN/LDAC	3/28 (10.7)	6/28 (21.4)	7/28 (25)	–	–	–	–	–	–	–	–

Valid percentages were calculated.

VEN: venetoclax; AZA: azacitidine; LDAC: low-dose cytarabine.

a 2017 European Leukemia Network (ELN) genetic risk stratification.

b Eight relapsed/refractory acute myeloid leukemia patients were included.

c Thirteen patients treated with VEN plus decitabine were included.

d Sixty-six patients treated with VEN plus decitabine were included, 45/169 (26.6) ELN inconclusive.

e Eighteen patients treated with VEN/AZA and ten patients treated with VEN/LDAC, 12/28 (42.9) ELN not determined.

### Somatic mutations

In seven of the studies, the frequency of common mutations such as *FLT3 ITD* (internal tandem duplications) was reported with a median of 14 % (range: 5.3–21.4 %);^15^^,^^17^, ^18^, ^19^^,^^21^, ^22^, ^23^ while the presence of *TP53* was reported in six studies with a median of 17.5 % (range: 16.7–42.8 %).^15^^,^^17^^,^^19^^,^^21^, ^22^, ^23^ Other reported mutations were *NPM1* with a median of 15.1 % (range: 7.9–42.8 %)^17^, ^18^, ^19^^,^^21^, ^22^, ^23^ and *IDH1* and *IDH2* in 14.2 % (range: 10.5–18.4 %)^17^^,^^18^^,^^22^ and 9.7 % (range: 2.6–16.8 %), respectively.^17^^,^^22^ The most frequently reported somatic mutation was *ASXL1* reported in five studies with a median of 25.3 % (range: 12.8–46.6 %).^15^^,^^17^^,^^21^, ^22^, ^23^ In two studies in the VEN/AZA group, *TP53* mutations were the predominant mutations with rates of 17.3 % ^21^ and 42.8 %,^19^ which is recognized as a poor prognostic factor regardless of cytogenetic profile.^27^ Regarding the *RUNX1* mutation, Cherry et al. reported that over 65-year-old patients with this mutation had a favorable outcome with the VEN/AZA combination (hazard ratio: 0.11; 95 % confidence interval [95 % CI]: 0.02–0.7; p-value = 0.01).^17^ Table 2 shows other clinically relevant mutations. In the study by Gomez-De Leon et al.,^25^ which included patients who received VEN/LDAC, the frequency of mutations was not reported.

### Adverse events

The incidence of adverse events was reported in four studies of patients receiving the VEN/AZA combination.^15^^,^^18^^,^^19^^,^^21^ In three of these studies, the most commonly reported Grade 3–4 adverse event was neutropenia with a median of 44.7 % (range: 28.5–70 %).^18^^,^^19^^,^^21^ In the study by Winters et al.,^15^ anemia was predominant with a frequency of 63.3 %, followed by neutropenia in 53.3 % of cases. Other adverse events reported were thrombocytopenia, anemia, nausea, and emesis.^15^^,^^18^^,^^19^^,^^21^ The study by Mirgh et al.^18^ was the only one that reported diarrhea (21 %) and neutropenic fever (32.7 %). The adverse events of VEN/LDAC^25^ were presented in conjunction with the data from patients who also received VEN/AZA, so we cannot discuss the adverse events of VEN/LDAC. Table 3 describes the adverse events of each study.

**Table 3:** Frequency of treatment-related adverse events (grade ≥3) of the 11 retrospective studies included in the systematic review.

First Author, Year	VEN/AZA and/or VEN/LDAC	Thrombocytopenia, *n* (%)	Neutropenia, *n* (%)	Anemia, *n* (%)	Náusea, *n* (%)	Vomiting, *n* (%)	Diarrhea, *n* (%)	Febrile neutropenia, *n* (%)
Winters et al., 2019	VEN/AZA	5/30 (16.7)	16/30 (53.3)	19/30 (63.3)	2/30 (6.7)	1/30 (3.3)	–	–
Abbott et al., 2020	VEN/AZA	–	–	–	–	–	–	–
Cherry et al., 2021	VEN/AZA	–	–	–	–	–	–	–
Mirgh et al., 2021 a	VEN/AZA	42/110 (38.2)	77/110 (70)	54/110 (49.1)	40/110 (36.4)	31/110 (28.2)	23/110 (21)	36/110 (32.7)
De Bellis et al., 2021 b	VEN/AZA	8/56 (14.3)	16/56 (28.5)	–	–	–	–	–
Jensen et al., 2022	VEN/AZA	–	–	–	–	–	–	–
Matthews et al., 2022	VEN/AZA	131/304 (43.1)	136/304 (44.7)	80/304 (26.3)	–	–	–	–
Garciaz et al., 2022	VEN/AZA	–	–	–	–	–	–	–
Mustafa Ali et al., 2022	VEN/AZA	–	–	–	–	–	–	–
Vachhani et al., 2022	VEN/AZA	–	–	–	–	–	–	–
Gómez-De León et al., 2022 c	VEN/AZA and VEN/LDAC	20/28 (71.4)	25/28 (89.3)	22/28 (78.6)	–	–	–	–

Valid percentages were calculated.

a Percentages calculated from 110 cycles administered, the sample includes eight relapsed/refractory acute myeloid leukemia patients.

b Thirteen patients treated with VEN plus decitabine were included.

c Eighteen patients treated with VEN/AZA and ten patients treated with VEN/LDAC.

## Discussion

Historically, for many years, before the introduction of hypomethylating agents, LDAC was the only medication for AML patients ineligible for chemotherapy; the benefits of LDAC were very limited in terms of OS. Subsequently, hypomethylating agents and VEN improved the OS curves.

In this systematic literature review of retrospective real-world studies, the lack of studies using the VEN/LDAC combination reflects the worldwide trend to favor the VEN/AZA combination over VEN/LDAC in the treatment of patients with AML who are not eligible for intensive therapy.^15^, ^16^, ^17^, ^18^, ^19^, ^20^, ^21^, ^22^, ^23^, ^24^, ^25^

In this review, we found only one study reporting the results of ten patients treated with VEN/LDAC, with a CCR rate of 90 % and a median OS not yet reached.^25^ This study had better results than the VIALE-C study,^3^ which had a CCR rate of 48 %, a CR rate of 18.8 %, and a median OS of 8.4 months. Perhaps these differences can be explained by the small sample size, short follow-up time, and the younger age of the patients in the study by Gómez-De León et al.^25^ We do not know the distribution of risk mutations in this study, or the distribution of treatment response based on cytogenetic risk, which may also have influenced the discrepancy in results.^25^ However, it is noteworthy that in this study, patients with VEN/LDAC had better CCR rates and OS than their VEN/AZA-treated counterparts (Table 1).

VIALE-C is the only Phase III clinical trial with a large number of patients providing information on the VEN/LDAC combination.^3^ However, it should be noted that VIALE-C included a patient population with unfavorable AML, particularly with regard to the incidence of secondary AML (41 %). In addition, prior exposure to hypomethylating agents was allowed, an exclusion criterion for the VIALE-A trial^2^; this is relevant because prior exposure to hypomethylating agents is associated with poor prognosis.^28^

As previously mentioned, the VIALE-A trial^2^ reported a median OS of 14.7 months, a CR rate of 36.7 %, and a CCR rate of 66.4 %. Compared with the results of the 11 retrospective VEN/AZA studies included in the present review, the median OS was 10.7 months, with a CR rate of 37.5 % and CCR of 63.3 %, thereby demonstrating similar results. In this context, it is worth noting that three studies^17^^,^^18^^,^^25^ reported higher CCR rates than VIALE-A.^2^ The first study was by Cherry et al.,^17^ which enrolled 143 patients on the VEN/AZA combination with a CCR rate of 71.3 %. The second study by Mirgh et al.^18^ included 16 patients and reported a CCR rate of 75 %, and the third study by Gomez-De Leon et al.^25^ enrolled 18 patients and reported a CCR rate of 72.2 %. The latter is noteworthy because the effect of clinical trials usually provides advantages in survival and response rates over real-world studies. A possible explanation for this could be differences between studies in the proportion of patients with favorable cytogenetic risk.

It is important to highlight that two of the reports with VEN/AZA included in the present review, one by Jensen et al.^20^ and the other by Garciaz et al.,^22^ reported OS curves of 9.1 and 9.4 months, respectively, which is similar to the OS curve of 8.4 months reported by the VIALE-C trial,^3^ but these reported OS curves are considerably shorter than the OS of 14.7 months reported in VIALE-A.^2^ This highlights the need to control for multiple variables in the study population to establish additional appropriate parameters and comparisons between these two regimens.

Qin Y et al.^29^ performed a meta-analysis on combinations of VEN with hypomethylating agents or LDAC as induction therapy for AML patients ineligible for intensive chemotherapy. They included only four clinical trials (three Phase Ib/II using VEN/hypomethylating agents and VIALE-C) and found pooled rates for CR of 40 % (95 % CI: 0.26–0.55) and CCR 64 % (95 % CI: 0.49–0.77) with a median OS of 11.7 (95 % CI: 10.15–14.18) months. It should be noted that the aim of this meta-analysis^29^ was to evaluate the efficacy and safety of VEN combinations and not to determine which VEN combination is more appropriate for a particular type of AML patient. Therefore, it was not possible to determine which combination (VEN/AZA or VEN/LDAC) showed greater benefit in isolation.

Even though, in this review, the most common cytogenetic risk category for the VEN/AZA combination was adverse (53.3 %) compared to VIALE-A and VIALE-C^2^^,^^3^ where the predominant category was intermediate, we highlight that some studies showed higher response rates compared to VIALE-A and even in the study by Cherry et al., a higher OS (16.1 months) was reported.^17^ We believe that this may be due to the proportion of included patients with favorable cytogenetic risk, which may have influenced the survival curves. We do not have cytogenetic risk data from real-world studies for the VEN/LDAC combination, so we can not analyze them.

It is noteworthy that in the VIALE-A trial,^2^ patients with favorable cytogenetic risk were excluded, resulting in reported distribution frequencies of 64 % and 36 % for the intermediate and adverse risk categories, respectively. The absence of favorable cytogenetic risk cases in the VIALE-A trial^2^ may explain why some real-world reports observed better response rates.

Regarding OS by cytogenetic risk, the VIALE-A trial reported a median OS for intermediate risk of 20.8 months, while for the adverse risk category the OS was 7.6 months.^2^ Other authors have pointed out that cytogenetic risk in conjunction with AML status (*de novo* versus secondary), age, and Eastern Cooperative Oncology Group (ECOG) scale functional status are independent baseline factors for OS.^3^

In the present work, we highlight that in the study by Cherry et al.,^17^ it was reported that the presence of *RUNX1* mutations was associated with a better prognosis in patients receiving the VEN/AZA combination compared with intensive chemotherapy. Similarly, the VIALE-A and VIALE-C trials reported that mutations with better prognosis (*IDH1/IDH2*) showed favorable outcomes with VEN/AZA or VEN/LDAC.^2^^,^^3^ Other authors have reported high response rates, durable remissions, and OS of up to 24.5 months for the VEN/AZA combination in patients with *IDH1/IDH2* mutations.^30^ On the other hand, patients included in both VIALE-A and VIALE-C, with poor prognostic mutations such as *TP53* and *FLT3*, showed no beneficial effect with any combination of VEN.^2^^,^^3^

It should also be noted that a subgroup analysis in the VIALE-C trial^3^ showed that the patients who benefited most were those with a *NMP1* mutation, with a CCR rate of 78 % and a median not reached OS. Interestingly, this mutation did not benefit from VEN/AZA as reported in the VIALE-A trial,^2^ although as we have mentioned, these are not formal direct comparisons. Based on these studies, we reason that the population that would benefit most from a formal comparison between AZA and LDAC in combination with VEN might be the favorable and intermediate-risk groups, including cases with the *IDH1–2* and *NPM1* mutations.

Hematologic toxicity, mainly neutropenia, was the most common adverse event in retrospective studies of the VEN/AZA or VEN/LDAC combinations, which is consistent with reports from the VIALE-A and VIALE-C studies.^2^^,^^3^ In VIALE-A, the main adverse events were nausea of any grade (44 %), febrile neutropenia (42 %), thrombocytopenia (45 %), and Grade 3 or higher neutropenia (42 %).^2^ In contrast, with VIALE-C, the main adverse events were nausea of any grade (42 %), febrile neutropenia (32 %), thrombocytopenia (45 %), and Grade 3 or higher (46 %) neutropenia.^3^ When comparing the safety profile, only the rate of febrile neutropenia is higher in the VEN/AZA group (42 %) than in the VEN/LDAC group (32 %). Currently, dose adjustment and the use of granulocyte colony-stimulating factor favor adequate management of neutropenia induced by VEN combinations.^31^ The study by Winters et al.^15^ was the only one that reported anemia as the main adverse event, followed by neutropenia. This may be due to the use of granulocyte colony growth factor in this patient cohort, which shortens the duration of febrile neutropenia induced by VEN.

Given the favorable response rates when VEN is combined with other drugs in patients with AML who are ineligible for intensive chemotherapy, VEN is currently considered the first-line treatment, regardless of their mutational status, except for the presence of a *TP53* mutation.^32^

To our knowledge, there are no direct comparisons of the efficacy of VEN/AZA and VEN/LDAC in newly diagnosed AML patients. However, one expert opinion publication^33^ states that the only scenario in which VEN/LDAC could be chosen instead of VEN/AZA is in patients with prior exposure to hypomethylating agents.

In this era of new pharmacological combinations, drug triplets including VEN^34^, ^35^, ^36^ are a therapeutic modality currently being investigated for the treatment of AML to overcome resistance in patients with poor prognosis, especially those with *TP53* mutations. In this scenario, cytarabine could be considered a viable combination option due to its known accessibility, low cost, efficacy, and toxicity profile.

The economic factor is another point to consider when deciding whether to combine VEN/AZA or VEN/LDAC. According to an economic analysis study, VEN/LDAC is less expensive than VEN/AZA ($75,833 versus $129,025 per year, respectively,^37^ and based on current costs, it has been indicated that the use of VEN/AZA is not affordable for all patients who need it, even in countries with developed economies.^11^ Accordingly, LDAC is significantly less expensive than AZA in Mexico ($300 versus $6500 per cycle).^25^ This may explain why in the publication by Gomez-De Leon et al.,^25^ VEN/LDAC was the most common combination in patients without insurance coverage.

## Conclusion

From the results of this review, it appears that the VEN/LDAC combination remains a second option to VEN/AZA for patients ineligible for intensive chemotherapy, and possibly an alternative treatment for refractory/relapsed AML in unfit patients, specifically after receiving hypomethylating agents.

However, it is important to consider that this inferiority to VEN/AZA was assumed based on the results of non-comparable studies designed for other purposes. Therefore, these studies have a less-than-optimal methodology for establishing the superiority of one treatment over another, which is an aspect that could be relevant to some subgroups of patients, such as patients with intermediate or favorable cytogenetic prognosis.

On the other hand, VEN/LDAC is useful for patients who have already been treated with hypomethylating agents or when drug cost is a critical factor to consider.

Comparative clinical trials between VEN/LDAC and VEN/AZA should be performed to properly classify each combination in the treatment of AML patients who are ineligible for intensive chemotherapy.

## Conflicts of interest

None.

## Appendix Supplementary materials

Image, application 1

## Footnotes

Supplementary material associated with this article can be found in the online version at doi:10.1016/j.htct.2024.07.006.
