# Supplementary material for: Venetoclax with low-dose cytarabine, a forgotten combination in patients with acute myeloid leukemia ineligible for intensive chemotherapy: a systematic review
Source: Hematol Transfus Cell Ther. 2024 Sep 23;46(Suppl 6):S322–31. doi: 10.1016/j.htct.2024.07.006 (PMC11726088; doi:10.1016/j.htct.2024.07.006)
Supplement: Supplementary file 1 [file mmc1.docx]

**Supplementary material**

**Table S1:** Inclusion and exclusion criteria for article screening

| **Type of included studies** | - Included: Real-world retrospective studies in English and Spanish published between January 1, 2015, and September 30, 2022 - Excluded: Abstracts, retrospective articles of clinical trials, and studies that did not separate the main outcomes from the target population in this review |
| --- | --- |
| **Condition or domain being studied** | - Acute myeloid leukemia |
| **Participants/population** | - Included: Adult (≥18 years old) patients with acute myeloid leukemia ineligible for intensive chemotherapy and on first-line treatment - Excluded: Relapsed or refractory acute myeloid leukemia patients and myelodysplastic diseases/myeloproliferative neoplasms patients |
| **Intervention(s), exposure(s)** | - Included: Therapeutic combination of venetoclax plus azacitidine or venetoclax plus low-dose cytarabine - Excluded: Therapeutic combination with a third or more drugs |
| **Comparator(s)/control** | - For those studies that included a control group treated with standard therapy or other drug combinations, these outcomes were not considered for the present review |
| **Main outcome** | - Overall survival (OS), complete remission (CR), and composite complete remission (CCR) |

**Table S2:** Search string for database searches

| **Search terms** | - **Study design:** retrospective study OR retrospectively OR cohort OR observational - **Population:** acute myeloid leukemia OR AML - **Standard treatment:** azacitidine OR azacytidine OR vidaza OR cytarabine OR cytosar OR aracytine - **Intervention:** venetoclax OR venclexta |
| --- | --- |
| **Time restriction** | - Studies published from January 1, 2015, to September 30, 2022 |
| **PubMed** | 1. (((((((((((((retrospective study[Title/Abstract]) OR (retrospectively[Title/Abstract])) OR (cohort[Title/Abstract])) OR (observational[Title/Abstract])) AND (acute myeloid leukemia[Title/Abstract])) OR (AML[Title/Abstract])) AND (azacitidine[Title/Abstract])) OR (azacytidine[Title/Abstract])) OR (vidaza[Title/Abstract])) OR (cytarabine[Title/Abstract])) OR (cytosar[Title/Abstract])) OR (aracytine[Title/Abstract])) AND (venetoclax[Title/Abstract])) OR (venclexta[Title/Abstract])   **Results:** 315 |
| **Web of Science** | 1. (((((((((((((TS=(retrospective study)) OR TS=(retrospectively)) OR TS=(cohort)) OR TS=(observational)) AND TS=(acute myeloid leukemia)) OR TS=(AML)) AND TS=(azacitidine)) OR TS=(azacytidine)) OR TS=(vidaza)) OR TS=(cytarabine)) OR TS=(cytosar)) OR TS=(aracytine)) AND TS=(venetoclax)) OR TS=(venclexta)   **Results:** 500 |

**Table S3:** Results of risk of bias assessment

| **The Newcastle-Ottawa Scale for quality assessment of observational studies.** | | | | | | | | | | | | |
| --- | --- | --- | --- | --- | --- | --- | --- | --- | --- | --- | --- | --- |
|  | | **Authors** | | | | | | | | | | |
|  | **Item** | Winters et al., 2019 | Abbott et al., 2020 | Cherry et al., 2021 | Mirgh et al., 2021 | De Bellis et al., 2021 | Jensen et al., 2022 | Matthews et al., 2022 | Garciaz  et al., 2022 | Mustafa et al., 2022 | Vachhani et al., 2022 | Gómez-De León et al., 2022 |
| **A** | **Selection** |  |  |  |  |  |  |  |  |  |  |  |
| 1) | Representativeness of the exposed cohort | - | - | - | - | 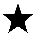 | 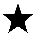 | 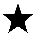 | - | - | 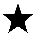 | 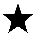 |
| 2) | Selection of the non-exposed cohort | 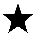 | 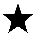 | 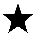 | - | - | 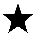 | 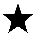 | - | 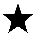 | - | - |
| 3) | Ascertainment of exposure | 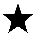 | 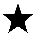 | 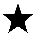 | 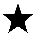 | 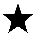 | 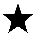 | 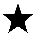 | 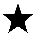 | 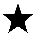 | 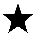 | 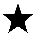 |
| 4) | Demonstration that outcome of interest was not present at the start of the study | 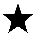 | - | 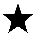 | 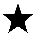 | 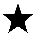 | 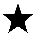 | 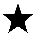 | 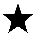 | 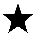 | - | 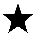 |
| **B** | **Comparability** |  |  |  |  |  |  |  |  |  |  |  |
| 1) | Comparability of cohorts on the basis of the design or analysis | - | - | 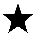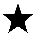 | - | - | 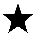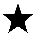 | 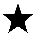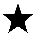 | - | 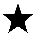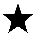 | - | - |
| **C** | **Outcome** |  |  |  |  |  |  |  |  |  |  |  |
| 1) | Assessment of outcome | 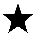 | 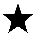 | 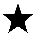 | 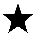 | 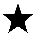 | 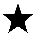 | 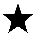 | 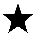 | 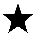 | 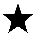 | 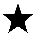 |
| 2) | Was follow-up long enough for outcomes to occur | 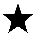 | 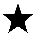 | 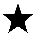 | 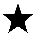 | 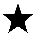 | 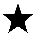 | 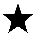 | 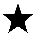 | 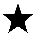 | 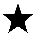 | 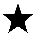 |
| 3) | Adequacy of follow-up of cohorts | 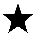 | 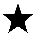 | 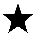 | - | - | - | 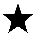 | - | 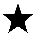 | - | - |
|  | **Total number of stars** | **6** | **5** | **8** | **4** | **5** | **8** | **9** | **4** | **8** | **4** | **5** |
|  | **Quality rating** |  |  |  |  |  |  |  |  |  |  |  |

A study can be awarded a maximum of one star for each of the items in the Selection and Outcome domains. In the case of the Comparability domain, each study can be awarded a maximum of two stars. Poor quality (red): 0 to 3 stars; Fair quality (yellow): 4 to 6 stars; Good quality (green): 7 to 9 stars.
